# Reconstruction of the Protein-Protein Interaction Network for Protein Complexes Identification by Walking on the Protein Pair Fingerprints Similarity Network

**DOI:** 10.3389/fgene.2018.00272

**Published:** 2018-07-24

**Authors:** Bo Xu, Yu Liu, Chi Lin, Jie Dong, Xiaoxia Liu, Zengyou He

**Affiliations:** ^1^School of Software, Dalian University of Technology, Dalian, China; ^2^Key Laboratory for Ubiquitous Network and Service Software of Liaoning, Dalian, China; ^3^School of Computer Science and Technology, Dalian University of Technology, Dalian, China

**Keywords:** protein complex, PPI network, network reconstruction, PPI prediction, bioinformatics

## Abstract

Identifying protein complexes from protein-protein interaction networks (PPINs) is important to understand the science of cellular organization and function. However, PPINs produced by high-throughput studies have high false discovery rate and only represent snapshot interaction information. Reconstructing higher quality PPINs is essential for protein complex identification. Here we present a Multi-Level PPINs reconstruction (MLPR) method for protein complexes detection. From existing PPINs, we generated full combinations of every two proteins. These protein pairs are represented as a vector which includes six different sources. Then the protein pairs with same vector are mapped to the same fingerprint ID. A fingerprint similarity network is constructed next, in which a vertex represents a protein pair fingerprint ID and each vertex is connected to its top 10 similar fingerprints by edges. After random walking on the fingerprints similarity network, each vertex got a score at the steady state. According to the score of protein pairs, we considered the top ranked ones as reliable PPI and the score as the weight of edge between two distinct proteins. Finally, we expanded clusters starting from seeded vertexes based on the new weighted reliable PPINs. Applying our method on the yeast PPINs, our algorithm achieved higher *F*-value in protein complexes detection than the-state-of-the-art methods. The interactions in our reconstructed PPI network have more significant biological relevance than the exiting PPI datasets, assessed by gene ontology. In addition, the performance of existing popular protein complexes detection methods are significantly improved on our reconstructed network.

## 1. Introduction

A protein complex is a group of associated polypeptide chains linked by noncovalent protein-protein interactions (PPIs). Protein complexes play important roles in biological systems and perform numerous biological functions, such as DNA transcription, mRNA translation, and signal transduction. Hence, identifying protein complexes in an organism is critical in molecular biology. With the advances of high-throughput technologies, many large-scale PPI networks have been constructed (Wan et al., 2015; Huttlin et al., 2017). Based on PPI information, *in silico* computational approaches have been developed to detect protein complexes, which has proven to be an effective approach to complement experimental methods for protein complex detection (Chen et al., 2014).

Computational approaches have been developed to identify protein complexes by searching densely connected regions in a PPI network (Li et al., 2010). The PPI network consists of nodes representing proteins and links representing physical interactions between a pair of proteins. The existing PPI netwoks are generally built using information gathered from high-throughput techniques mentioned above, which have many errors and missing information (Huttlin et al., 2017). It has a high false positive rate and even a higher false negative rate (Wan et al., 2015). Detecting protein complexes from these protein interaction networks has been limited in accuracy due to these false interactions. Many recent studies integrated other functional information into the protein interaction networks to accurate the PPINs for improving the performance of protein complexes detection (Chen et al., 2014). For example, a graph fragmentation algorithm incorporated microarray gene expression profiles to help refine the putative complexes (Feng et al., 2011). Zeng et al. (2016) presented a features fusion method which used n-gram frequency method to extract features based on protein sequence to improve the prediction. Jung et al. (2010) presented a simultaneous protein interaction network, which removed the mutually exclusive interactions based on domain information. Xu et al. (2011) generated weighted PPI networks based on semantic similarity of each protein pair in the Gene Ontology (GO). CMC (clustering based on maximal cliques) (Liu et al., 2009) used an iterative scoring method to assign a weight to protein pairs, which indicated the reliability of the interaction between the two proteins. Krogan et al. (2006) assigned a reliability score to every protein pair by converting multirelationships in the AP-MS data into binary interactions for predicting protein complexes. All these existing methods try to accurate the PPI network with some other biological or topological evidence for protein complex identification. However, these methods only resolve the false positives of PPINs and only 1 or 2 PPI evidences are used in these processes. Therefore, more effort needs to be devoted toward improving the quality of the existing PPI networks for protein complexes identification.

**Figure 1:**
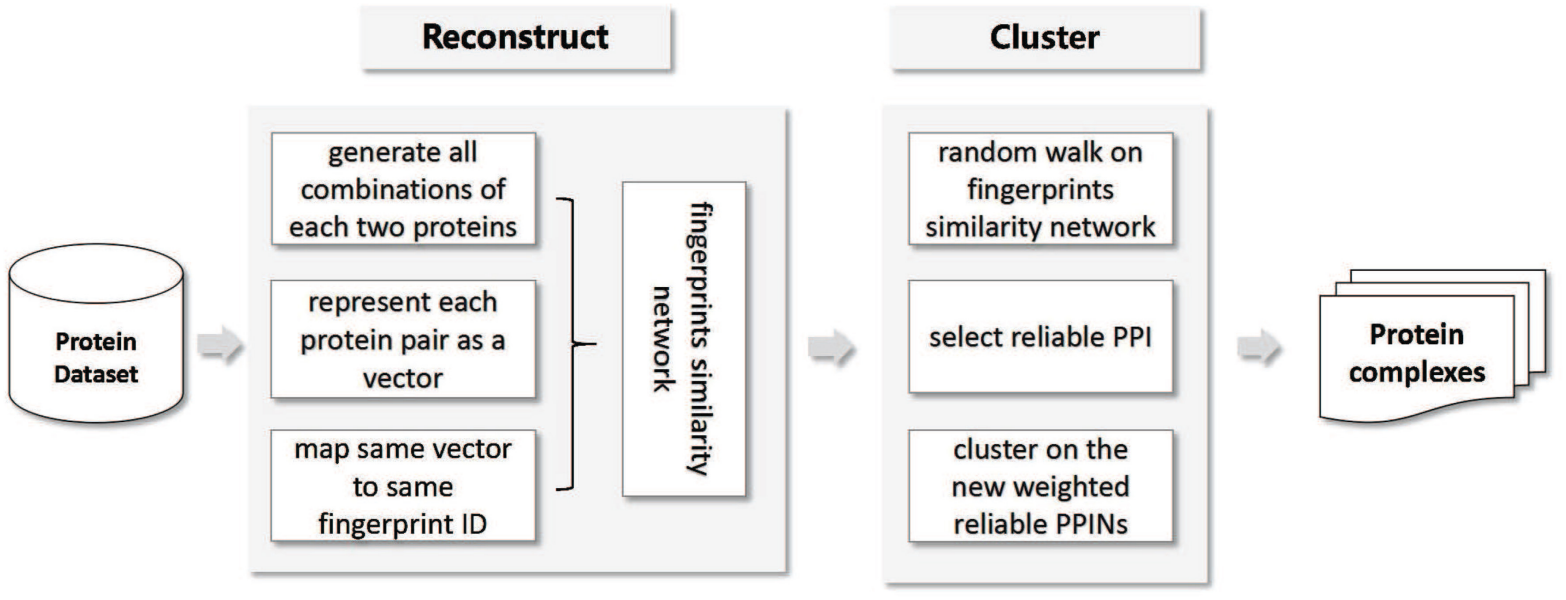
The working flow of our method.

In this paper, we proposed a Multi-Level PPINs reconstruction (MLPR) method to remove spurious protein interactions and recover missing ones for protein complexes identification. First, we generated all combinations of each two proteins and represented each protein pair as a vector which included 17 features gathered from six sources (Gene Ontology, Gene expression, Domain-Domain Interaction, String, AP-MS experiment, PPI network properties). Second, protein pairs with same vector are mapped to an ID which is called protein pair fingerprint ID. Each fingerprint ID represents a set of protein pairs which have same vector. Third, a fingerprint-similarity network is constructed, in which a vertex represented a fingerprint and an edge represented the similarity between two distinct fingerprints. Forth, we performed a random walk with restart algorithm on this fingerprints similarity network. Some fingerprints of reliable protein interactions are given prior probabilities 1. At the end of the iterations, every fingerprint reached a steady state and got a probability. The protein pairs are selected as reliable PPI whereby the corresponding fingerprints probability from random walk algorithm. Finally, we expanded clusters starting from seeded vertexes based on the new weighted reliable PPINs for identifying protein complexes. Figure 1 shows the flowchart of our method.

## 2. Methods

For a given organism, the proposed protein complex identification approach contains two steps. The first step is to reconstruct a high quality PPI network by removing spurious interactions and recover missing ones. The second step is to expand clusters starting from seeded vertexes based on the new weighted reliable PPINs for identifying protein complexes. Here, we first describe Multi-Level PPINs reconstruction approach for getting reliable PPI and then present the detailed protein complexes identification method on the new reliable PPINs.

### 2.1. Reconstruction of a PPI network by random walking on the protein pair fingerprints similarity network

**Figure 2:**
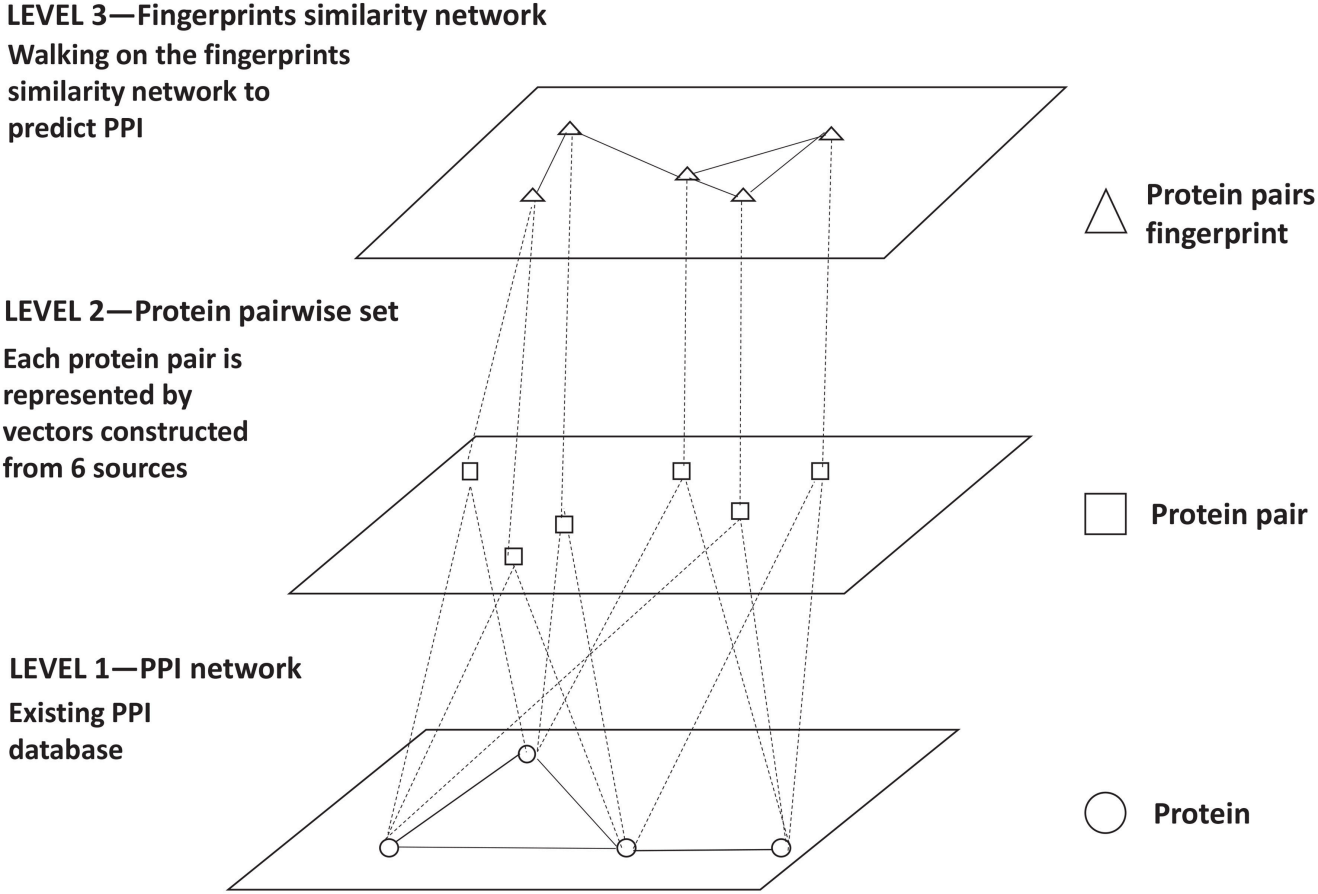
High-level reconstructed network. The first level is the existing PPI networks. The second level is the protein pairs annotated with six sources. The third level is the protein pair fingerprints similarity network.

Existing PPI datasets are transferred to a protein pair fingerprint similarity network for getting reliable PPI (Figure 2). We first generated all combinations of each two proteins in the existing networks (Level 1) and represented each protein pair as a vector which included *n* features gathered from *m* sources (Level 2). Consequently, protein pairs represented by same vector were mapped to same fingerprint ID. A fingerprint similarity network is constructed, in which a vertex represents a protein pair fingerprint ID and each vertex is connected to its top *t* similar fingerprints by edges (Level 3). Then we performed a random walk with restart algorithm on this fingerprints similar network. Some fingerprints of reliable protein interactions are given prior probabilities 1. At the end of the iterations, every fingerprint reached a steady state and got a probability. The steady state probability of each fingerprint is the probability of corresponding protein pairs to be a reliable PPI. The top ranked protein pairs are selected as reliable PPI. The details are described below.

#### 2.1.1. Protein pairs with PPI evidences

Following our previous method (Xu et al., 2013), our approach is to characterize each protein pair using PPI evidences from multiple sources. The multiple sources include Domain-Domain interaction (D), molecular function (MF) of GO, biological processes (BP) of GO, cellular components (CC) of GO, gene co-expression (CE), STRING (S), TAP-MS (TAP), existing PPI database (EPPI), as well as the proteins' corresponding topological properties in the existing PPI networks (CD). These features are listed below.

##### 2.1.1.1. Gene ontology annotations

GO (Ashburner et al., 2000) is a framework for the model of biology that defines concepts used to describe gene function, and relationships between these concepts. It contains three aspects that hold terms defining the basic concepts of molecular function (MF), biological processes (BP), and cellular components (CC), respectively. GO terms are arranged in directed acyclic graphs. GO slims are cut-down versions of the GO ontologies containing a subset of the terms in the whole GO. They give a broad overview of the ontology content without the detail of the specific fine grained terms. GO slims give a comprehensive description of proteins biological attributes. A protein pair has a high probability of being a PPI pair when they have similar GO annotations. We used two different types of measures to calculate the similarity of GO annotations for a protein pair. One type (Type I) uses the semantic similarity measure of Lord et al. (2003). It is based on the hypothesis that a term is more informative if it and its descendants have fewer annotated genes or proteins in an ontology. The other type (Type II) is based on organism-specific GO Slims. Given a protein pair, the similarity value is defined as 1 if two proteins shared at least 1 common GO Slim term after removing trivial root GO terms; otherwise, the value is 0. The GO website was accessed in September 2011 to retrieve GO annotations and GO Slim terms for yeast. A total of six features were defined by combining the two similarity types and the three aspects (*MF, mf, BP, bp, CC, cc*).

##### 2.1.1.2. Gene coexpression

The corresponding genes of the proteins in a protein complex are expected to be coexpressed (i.e., activated and repressed under the same conditions) (Jansen et al., 2003; Bhardwaj and Lu, 2005; Li et al., 2006). To capture gene coexpression information of a protein pair, we defined a feature by using many microarray data series available in Gene Expression Omnibus (Edgar et al., 2002). For that we downloaded a total of 161 microarray data series for yeast (using platform PL90), consisting of 2,015 samples, from Gene Expression Omnibus (accessed September 2011). The expression measures were log transformed, and a Pearson correlation coefficient was computed as a feature (*CE*) for each protein pair.

##### 2.1.1.3. Domain-domain interaction

A protein domain is a conserved part of a given protein sequence and structure that can evolve, function and exist independently of the rest of the protein chain. Many proteins consist of several structural domains. Domains often suggest the propensity for the proteins to interact or form a functional unit, such as protein complex. So we used one feature to capture Domain-Domain interaction (DDI) information for a protein pair. The domains (Pfam) of yeast proteins were downloaded from UniProtKB (Apweiler et al., 2004). The Domain-Domain interaction (DDI) information were downloaded from InterDom (Ng et al., 2003), in which each DDI pair is assigned a confidence score. And the value of a DDI feature (*D*) for a protein pair was set as the sum of the confidence scores of all possible DDI pairs between them.

##### 2.1.1.4. STRING evidence

STRING (Jensen et al., 2009) is a database of known and predicted protein-protein interactions. The interactions include direct (physical) and indirect (functional) associations; they stem from computational prediction, from knowledge transfer between organisms, and from interactions aggregated from other (primary) databases. So it is an essential source for our work. To indicate the confidence of PPI, a score is assigned by STRING for each protein pair. We used that score as the feature (*S*) to capture STRING-predicted evidence of PPI information.

##### 2.1.1.5. AP-MS experiments

The high-throughput AP-MS experiments have generated a large amount of bait-prey data, posing great challenges on the computational analysis of such data for inferring true interactions and protein complexes. Many computational methods have been developed to detect true protein complexes from AP-MS data. These methods typically convert the co-complex relationships in the AP-MS data into binary PPIs. They proposed different measurements to assign a reliability score to every protein pair. The higher the scores are, the more reliable of the candidate PPIs. These scores of PPIs are powerful information for protein complexes detection. Here we downloaded the candidate PPIs with reliable score form Krogan core (*TAP*1) and extended (*TAP*2) data (Krogan et al., 2006), Hart (*TAP*3) (Hart et al., 2007), Gavin (*TAP*4) (Gavin et al., 2006), and Collins (*TAP*5) (Collins et al., 2007). We used those scores directly as TAP features.

##### 2.1.1.6. PPI network properties

Not every interaction pair is presented in accurated PPI networks. We used two types of evidence to capture existing PPI network information. Type I is the direct information from existing PPI data. If one pair is recorded in one exiting PPI data, its EPPI value is equal to 1, otherwise, the value was 0. We downloaded yeast protein interaction data from DIP (Xenarios et al., 2002) and BioGRID (Stark et al., 2006) as this Type I features. Type II is the indirect information from PPI network topology. We consider a protein pair to have a higher probability of being a PPI pair if they have many common neighbors in a PPI network. We use the Czekanowski-Dice distance (Brun et al., 2003; Chen et al., 2006) (CD-distance) based on DIP to capture such information (*CD*).

As described above, each protein pair *P*_*i*_ is represented as a vector, *V*_*pi*_ which consists of a domain component *D*_*pi*_, molecular function in GO terms and GO Slims components *MF*_*pi*_ and *mf*_*pi*_, biological process in GO terms and GO Slims components *BP*_*pi*_ and *bp*_*pi*_, cellular component in GO terms and GO Slims components *CC*_*pi*_ and *cc*_*pi*_, gene co-expression component *CE*_*pi*_, STRING component *S*_*pi*_, PPI reliable score based on TAP-MS from Krogan core, Krogan extended, Hart, Gavin, and Collins components *TAP*1_*pi*_, *TAP*2_*pi*_, *TAP*3_*pi*_, *TAP*4_*pi*_, and *TAP*5_*pi*_, existing PPI databases BioGRID and DIP components *EPPI*1_*pi*_, *EPPI*2_*pi*_, and PPI topological in DIP component *CD*_*pi*_, i.e., *V*_*pi*_ = (*D*_*pi*_, *MF*_*pi*_, *mf*_*pi*_, *BP*_*pi*_, *bp*_*pi*_, *CC*_*pi*_, *cc*_*pi*_, *CE*_*pi*_, *S*_*pi*_, *TAP*1_*pi*_, *TAP*2_*pi*_, *TAP*3_*pi*_, *TAP*4_*pi*_, *TAP*5_*pi*_, *EPPI*1_*pi*_, *EPPI*2_*pi*_, *CD*_*pi*_). *MF*_*pi*_, *BP*_*pi*_, *CC*_*pi*_ are boolean vectors and the others are numeric vectors.

#### 2.1.2. Protein pair fingerprints similarity network

A PPI network is constructed from existing PPI knowledge by considering individual proteins as nodes and the existence of a physical interaction between a pair of proteins as a link. Based on the nodes in these existing PPI networks, full combinations of every two nodes are generated. These generated protein pairs are represented by the vectors as described above. For reducing computational complexity, the protein pairs with same vector are mapped to the same fingerprint ID. So each fingerprint represents a set of protein pairs and it is also represented by the corresponding vector. Then a fingerprint similarity network *F*_*sim*_ = (*V*_*sim*_, *E*_*sim*_) is constructed, in which a vertex *v* in vertex set *V*_*sim*_ represents a fingerprint *f*_*i*_ and an edge (*f*_*i*_, *f*_*g*_) in edge set *E*_*sim*_ represents a connection between two distinct fingerprints *f*_*i*_ and *f*_*j*_. To construct *F*_*sim*_, we define the fingerprints pairwise similarity matrix *M*_*ij*_ between any two fingerprints *f*_*i*_ and *f*_*j*_ as follows:

(1) $$\begin{array}{l} M_{ij}=1-\frac{dist\left(f_{i},f_{j}\right)\;-\;min_{v\in V_{sim}}dist\left(f_{i},f_{j}\right)}{max_{v\in V_{sim}}dist\left(f_{i},f_{j}\right)\;-\;min_{v\in V_{sim}}dist\left(f_{i},f_{j}\right)}, \end{array}$$

where *dist*(*f*_*i*_, *f*_*j*_) is the Euclidean distance. A high value in *M*_*ij*_ indicates that the two fingerprints *f*_*i*_ and *f*_*j*_ share the similar PPI evidences and thus likely belong to same category (PPI or non-PPI). For each fingerprint *f*_*i*_ ∈ *V*_*sim*_, we connect it with another fingerprint if their similarities are among top *T* similar ones to fingerprint *f*_*i*_.

#### 2.1.3. Walking on the protein pair similarity network

With the above resulting protein pair fingerprints similarity network *F*_*sim*_ = (*V*_*sim*_, *E*_*sim*_), we can then perform a random walk with restart algorithm to detect the likely reliable PPI fingerprints and unreliable PPI fingerprints as below.

We first initialize the prior probabilities of fingerprints. The fingerprint is considered as reliable PPI fingerprint if it is from at least two accurated PPI database and above half PPI evidence components are non-zero. The other fingerprints are considered as unknown fingerprints. Let *R*_0_ and *U*_0_ denote the prior probability vector of the reliable and unknown fingerprints, respectively. In *R*_0_, the prior probabilities of reliable fingerprints are assigned an equal probability +1. This is equivalent to letting the random walk begin from each of reliable PPI fingerprints with equal probability. In *U*_0_, the prior probabilities of unknown fingerprints are assigned 0 and their posterior probabilities will be decided in step 2. We represent the overall prior probability vector for the fingerprints similarity network as $F_{0}={\left(R_{0},U_{0}\right)}^{T}$.

After initialing the prior probabilities for reliable and unknown examples above, we score all the remaining unknown fingerprints in the network by transmission. We propose to do flow propagation for this and adopt the Random Walk algorithm (Lovász et al., 1993) to our network *F*_*sim*_. The prior influence flows of reliable fingerprints are distributed to their neighbors, which continue to spread the influence flows to other nodes iteratively. Here, we used a variant of the random walk in which we additionally allow the restart of the walk in every step at one node with probability. Formally, the random walk with restart is defined as:

(2) $$\begin{array}{l} F_{r}=\left(1-\alpha \right)M_{ij}F_{r-1}+\alpha F_{0},\left(r\geq 2\right), \end{array}$$

where *F*_0_ is the initial probability vector, *F*_*r*_ is the probability vector at step *r*, *F*_1_ = *F*_0_, *M*_*ij*_ is row-normalized adjacency matrix of the graph. In this work we set parameter to 0.8, as recommend in Li and Patra (2010). At the end of the iterations, the prior information held by every vertex in the network will reach a steady state as proven by Lovász et al. (1993). This is determined by the probability difference between *F*_*r*_ and *F*_*r*−1_, represented as *D*_*if*_ = |*F*_*r*_ − *F*_*r*−1_|(measured by *L*1 norm). When *D*_*if*_ fell below 10^−6^, a steady stage has been reached and the iterative process is terminated.

According to the posterior probabilities of *U*_0_, we further select some likely reliable PPI fingerprints. Protein pair sets corresponding to the selected fingerprints, each protein pair gets a score. The high rank protein pairs are considered as the reliable ones.

### 2.2. Identifying protein complex from the new reliable PPINs

Motivated by previous methods (Li et al., 2008; Xu et al., 2011), we also expanded clusters starting from seeded vertexes. While the weighted vertexes and selecting seed are based on our new reliable PPI network. As mentioned above, the reliable score of PPI is the weight of the edge between two proteins. We define the weight of each vertex to be the sum of the weights of its incident edges. After all vertexes are assigned weights, we also sort the vertexes in non-increasing order by their weights and store them in a queue *S*_*q*_ (vertexes of the same weight are ordered in terms of their degrees). Here, we also pick the highest weighted vertexes as the seeds. Our procedure proceeds as follows. We pick the first vertex in the queue *S*_*q*_ and use it as a seed to grow a new cluster. Once the cluster is completed, all vertexes in the cluster are removed from the queue *S*_*q*_ and we pick the first vertex remaining in the queue *S*_*q*_ as the seed for the next cluster.

We also used *E*_*vk*_ to measure how strongly a vertex *v* is connected to a subgraph *K*: the interaction probability *E*_*vk*_ of a vertex *v* to a subgraph *K*, where *v* ∉ *K*, is defined by

(3) $$\begin{array}{l} E_{vk}=\frac{e_{vk}}{w_{k}}, \end{array}$$

where *e*_*vk*_ is the sum of the weights of edges between the vertex *v* and *K*, and *w*_*k*_ is the sum of weights of edges in *K*. A cluster *K* is extended by adding vertexes recursively from its neighbors according to the priority. The priority of a neighbor *v* of *K* is determined by the value *E*_*vk*_.

Let *T*_*in*_ be a threshold ranging between 0 and 1, let *d* be a positive integer, and let *K* be a subgraph. *SP* is the shortest path. A vertex *v* ∉ *K* is added to the cluster if the following two conditions are satisfied (where *K* + *v* denotes the subgraph induced by *K* and *v*):

1. *E*_*vk*_ ≥ *T*_*in*_; *and*
2. *The*(*SP*(*K* + *v*) ≤ *d*)

Only when the candidate vertex *v* is satisfied the conditions, can it be added to the cluster. Once the new vertex *v* is added to the cluster, the cluster is updated.

## 3. Results

### 3.1. Experimental data

We downloaded 7,018 yeast proteins from the Saccharomyces Genome Database (Cherry et al., 1998) and generated 24.6 million protein pairs. We also downloaded yeast protein interaction data from DIP (Xenarios et al., 2002), BioGRID (Stark et al., 2006), Krogan core and extended data (Krogan et al., 2006), Hart (Hart et al., 2007), Gavin (Gavin et al., 2006) and Collins (Collins et al., 2007) to evaluate our method. The details of these datasets are shown in Table 1. The yeast protein complex data were downloaded from a public repository (http://wodaklab.org/cyc2008/) with a total of 408 manually accurated heteromeric protein complexes. After filtering out complexes composed of a single or a pair of proteins, the final benchmark set contains a total of 231 protein complexes.

**Table 1:** The basic statistical information of different datasets.

**PPI networks**	**Number of proteins**	**Number of interactions**
BioGRID	5,640	59,748
Collins	1,622	9,074
DIP	4,928	17,201
Gavin	1,430	6,531
KroganCore	2,708	7,123
KroganExtended	3,672	14,317

### 3.2. Performance evaluation

We applied three approaches (Min et al., 2009) to evaluate the experimental performance. Equation (4) calculates the neighborhood affinity score *NA*(*p, b*) between a predicted cluster *p* ∈ *P* and a real complex *b* ∈ *B*, where *P* is the set of predicted complexes by a computational method and *B* is the set of positive ones in the benchmark.

(4) $$\begin{array}{l} NA\left(p,b\right)=\frac{|V_{p}⋂V_{b}{|}^{2}}{\left|V_{p}|\times |V_{b}\right|}. \end{array}$$

In Equation (4), |*V*_*p*_| is the number of proteins in the predicted complex and |*V*_*b*_| is the number of proteins in the real complex. If *NA*(*p, b*) ≥ ω, a real complex and a predicted complex are considered to be matching (ω is usually set as 0.20 or 0.25) (Bhowmick and Seah, 2016). After all real complexes and predicted clusters have their best match calculated according to their *NA* scores, *precision*, *recall*, and *F*-*value* are applied to assess the methods:

(5) $$\begin{array}{l} N_{cp}=|\left\{p|p\in P,\exists b\in B,NA\left(p,b\right)\geq \omega \right\}|, \end{array}$$

(6) $$\begin{array}{l} N_{cb}=|\left\{b|b\in B,\exists p\in P,NA\left(p,b\right)\geq \omega \right\}|, \end{array}$$

(7) $$\begin{array}{l} Precision=\frac{N_{cp}}{\left|P\right|},Recall=\frac{N_{cb}}{\left|B\right|}, \end{array}$$

(8) $$\begin{array}{l} F\text{-}value=2\times Precision\times Recall/\left(Precision+Recall\right). \end{array}$$

*N*_*cp*_ is the number of predicted complexes that match at least one real complex, and *N*_*cb*_ is the number of real complexes that match at least one predicted complex (Bhowmick and Seah, 2016).

#### 3.2.1. *P*-value (functional homogeneity)

The statistical significance of the occurrence of a protein cluster (predicted protein complex) with respect to given functional annotation can be computed by the following hypergeometric distribution in Equation (9) (Li et al., 2010):

(9) $$\begin{array}{l} P-value=1-\overset{k-1}{\underset{i=0}{\sum }}\frac{\left(\begin{array}{c} \left|F\right|\\ i \end{array}\right)\left(\begin{array}{c} \left|V|-|F\right|\\ |C|-i \end{array}\right)}{\left(\begin{array}{c} \left|V\right|\\ \left|C\right| \end{array}\right)}. \end{array}$$

where a predicted complex *C* contains *k* proteins in the functional group *F* and the whole PPI network contains |*V*| proteins. The functional homogeneity of a predicted complex is the smallest *P*-*value* over all the possible functional groups. A predicted complex with a low functional homogeneity indicates it is enriched by proteins from the same function group and it is thus likely to be true protein complex.

### 3.3. Evaluation of reconstructed PPINs

From the Saccharomyces Genome Database (Cherry et al., 1998), we generated 24.6 million protein pairs (all combinations of each two proteins). Each protein pair is represented as a vector which includes 17 features from six sources. The protein pairs with same vector are mapped to the same fingerprint ID. A total of 1,200,147 fingerprints are generated. So a fingerprint represented a set of protein pairs and is also considered as the same vector with the corresponding protein pairs. For each fingerprint, the top ten similar fingerprints have edges linked to it. The random walking algorithm is then performed on the fingerprints similarity network. The fingerprints prior probability is set to 1 if their *TAP*3 or *TAP*5 value is equal to 1 (recorded in Krogan core or Collins datasets) and more than half PPI evidence components are non-zero. After random walking on the fingerprints similarity network, each fingerprint has a posterior probability.

According to this fingerprints' posterior probability, each protein pair has a corresponding score, in which the score measures the possibility or confidence of a pair to be reliable PPI. We then ranked the pairs by the scores, and those high ranked ones were considered to be reliable PPI pairs.

To evaluate our reconstructed PPI network, we performed a statistical analysis for our predicted PPIs based on GO annotations. We compared different edge groups for the functional relevance between nodes connected by an edge. The hypothesis is that if our algorithm reduces noise in the PPI network, the edges in our networks are functionally more relevant than other networks. Since interacting proteins are likely involved in similar biological processes, they are expected to have similar functional annotations in gene ontology. Therefore, we measure the functional relevance between any pair of genes that are connected by an edge using the semantic similarity between the GO terms annotated with the proteins, using a popular method (Lord et al., 2003). Experimental results show that the proportion of PPIs in one network whose similarity is above 0.5 in three branches of GO (BP, CC, MF) (Table 2). As the number of selected PPI increases, the relevance decreases slightly. But they are still higher than PPI in BioGRID, DIP, Gavin, Krogancore, and Kroganextened datasets. The relevence of top 9,000 PPI is even higher than that of Collins. All these indicate that our method get a higher quality network for protein complexes detection.

**Table 2:** The relevance of Protein pairs in different datasets.

	**CC**	**BP**	**MF**
TOP6000	0.995667	0.994168	0.812531
TOP7000	0.991143	0.992	0.798143
TOP8000	0.98588	0.989379	0.786205
TOP9000	0.977005	0.985892	0.782048
TOP10000	0.9651	0.9779	0.778
TOP11000	0.956455	0.970909	0.773364
TOP12000	0.951083	0.967	0.757
TOP13000	0.942385	0.958692	0.742077
TOP14000	0.933286	0.949429	0.728571
TOP15000	0.9256	0.941133	0.7178
TOP16000	0.917625	0.933063	0.710625
BioGRID	0.782369	0.816847	0.593902
Collins	0.96793	0.971126	0.73672
DIP	0.791407	0.740771	0.541248
Gavin	0.904942	0.897901	0.656148
KroganCore	0.83083	0.834901	0.603959
KroganExtended	0.783614	0.802542	0.579613

**Figure 3:**
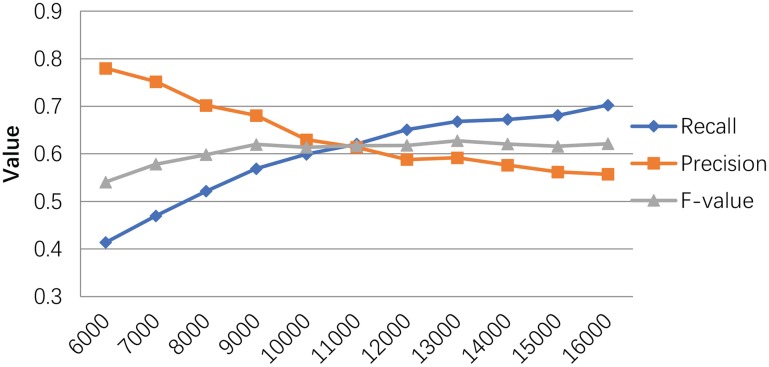
The performance of our MLPR method on our reconstructed PPINs.

We also evaluated our method based on different size reconstructed networks. The *T*_*in*_ is set to 0.6 for our experiments. Figure 3 shows the trend of our method's performances when selecting different network sizes. Generally, the recall rate increases when the number of predicted PPI pairs increases. The precision rate slightly decreases as the network size increases. While the *F*-*value* goes up with the network size increases and reaches its peak around 13,000.

**Figure 4:**
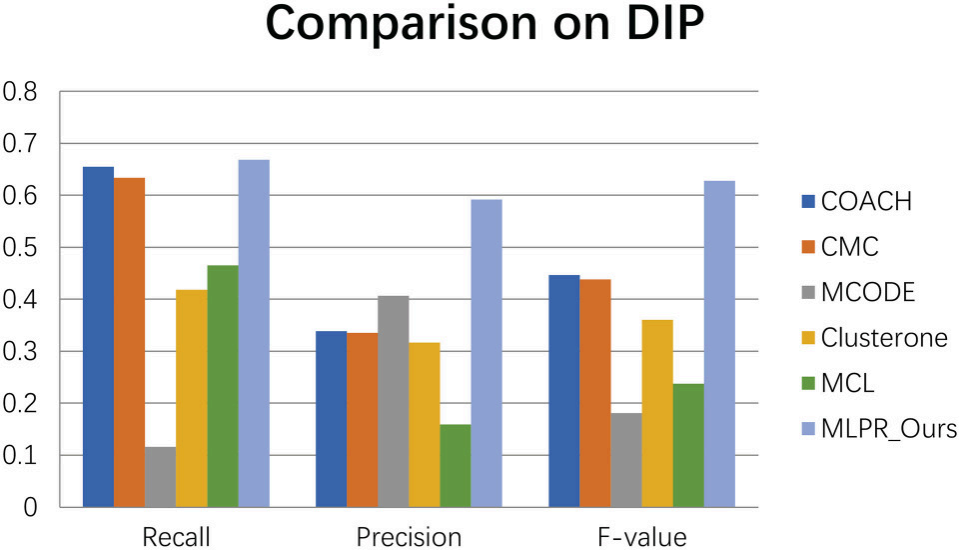
The performance of our MLPR method on our reconstructed PPINs.

**Figure 5:**
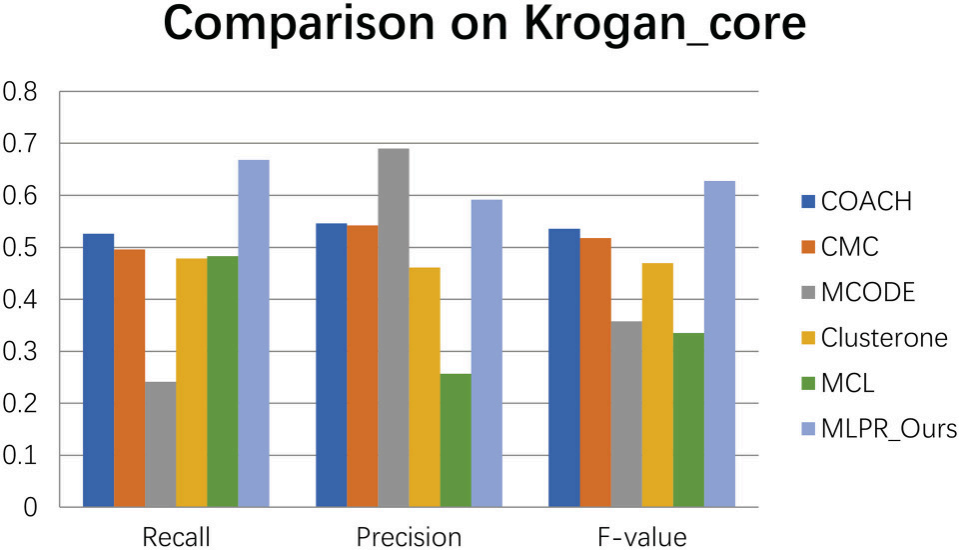
The performances comparison between our method and other five methods on Krogan core dataset.

**Figure 6:**
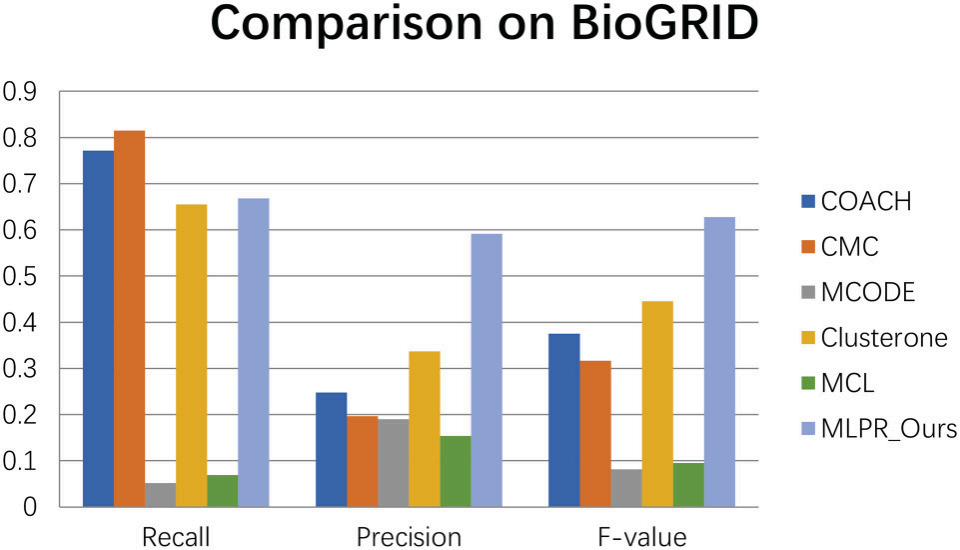
The performances comparison between our method and other five methods on BioGRID dataset.

**Figure 7:**
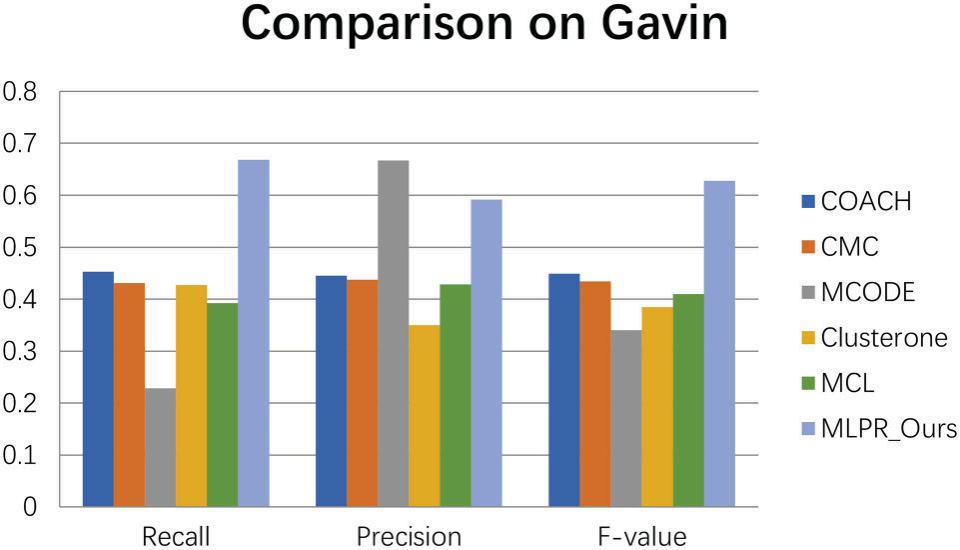
The performances comparison between our method and other five methods on Gavin dataset.

**Figure 8:**
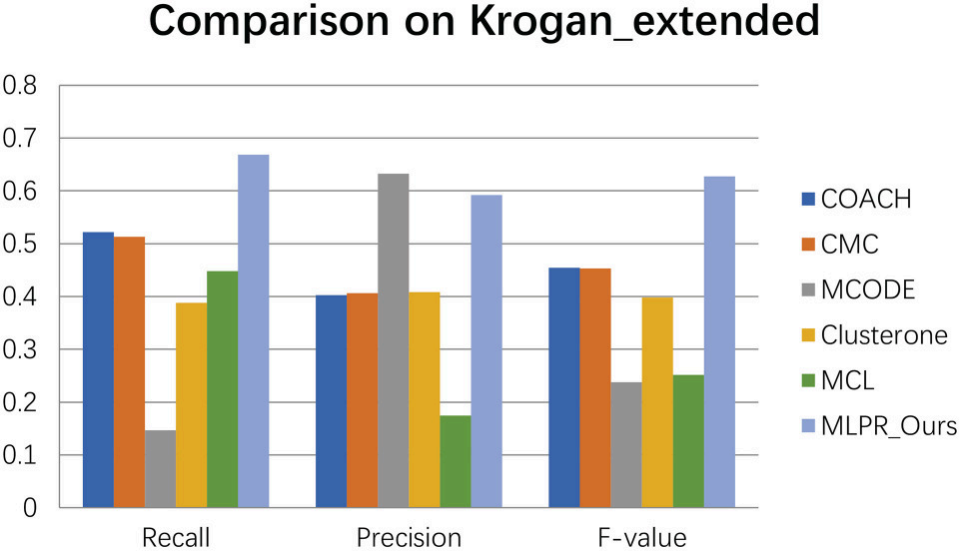
The performances comparison between our method and other five methods on Krogan extended dataset.

**Figure 9:**
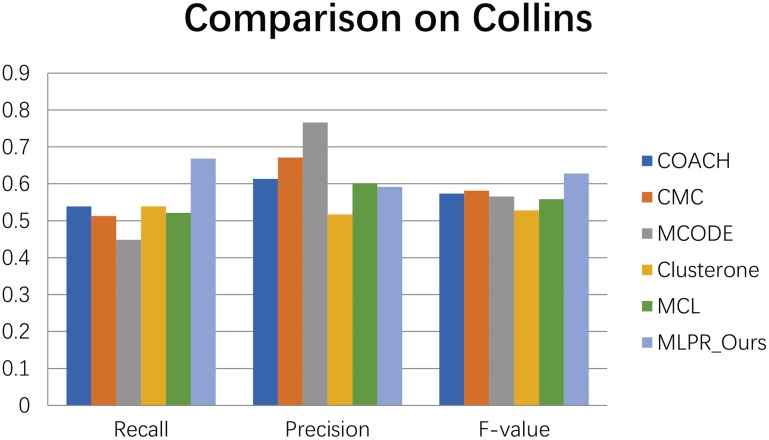
The performances comparison between our method and other five methods on Collins dataset.

We compared our method with the existing popular protein complexes detection methods including COACH (Min et al., 2009), CMC (Liu et al., 2009), MCODE (Bader and Hogue, 2003), Clusterone (Nepusz et al., 2015), and MCL (Van Dongen, 2000) on different networks. The parameters of these methods are set to default values as mentioned in their original papers. They are implemented on the existing PPI networks DIP (Xenarios et al., 2002), BioGRID (Stark et al., 2006), Gavin (Gavin et al., 2006), Collins (Collins et al., 2007), and Krogan core and extended (Krogan et al., 2006) respectively. As shown in Figures 4–9, our method MLPR achieved higher *F*-*value* than other methods on the six PPI networks. We also achieved higher *Recall* on DIP, Gavin, Collins, Krogan core, and extended PPI networks except on BioGRID. But we achieved a higher *Precision* than other methods on BioGRID. All this indicates that our method enhance the performance of protein complexes detection algorithms.

**Figure 10:**
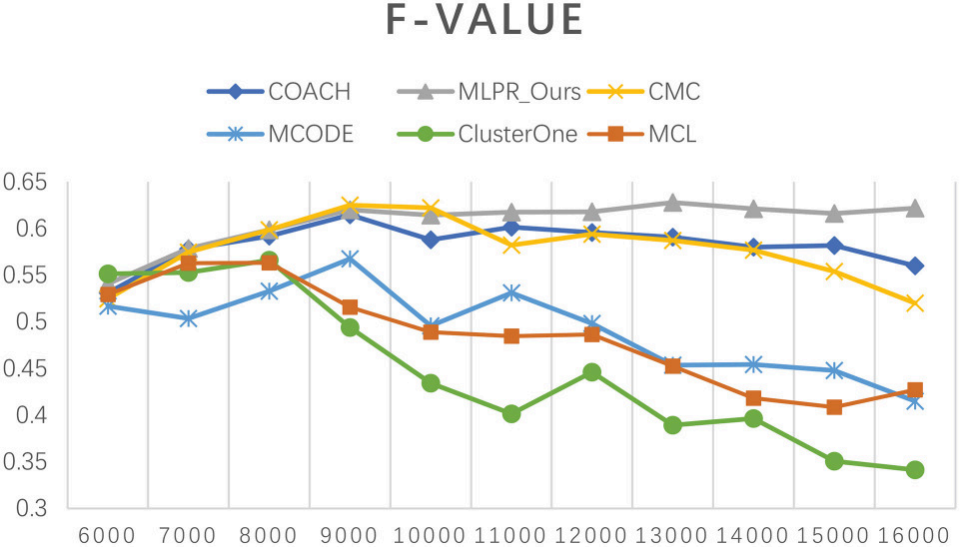
The *F*-value of our method and other five methods on our reconstructed networks.

**Figure 11:**
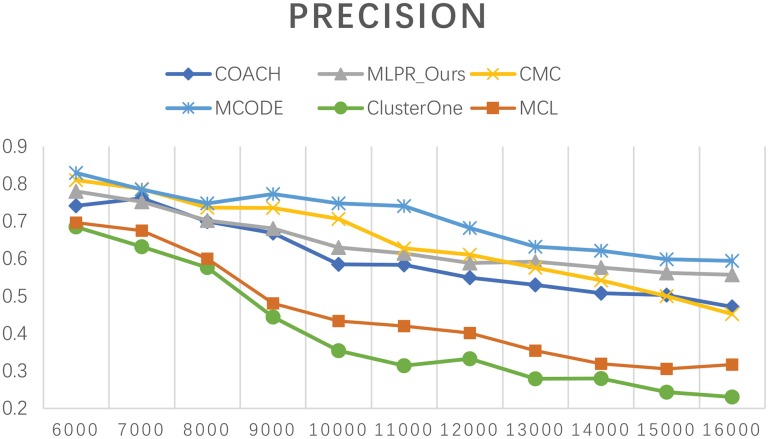
The precision of our method and other five methods on our reconstructed networks.

**Figure 12:**
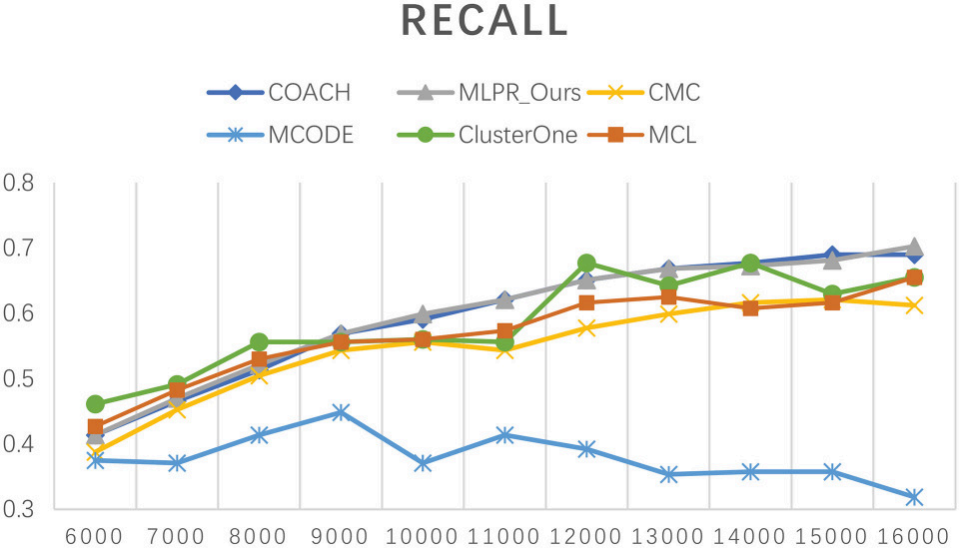
The recall of our method and other five methods on our reconstructed networks.

Besides comparing our method with others on the six existing PPI network, we also employed COACH, CMC, MCODE, Clusterone, and MCL on our reconstructed PPI network. Figures 10–12 show the trend of methods' performance when selected different size networks that reconstructed with the top 6,000–16,000 predicted reliable PPI pairs. The recall rate increases when the number of predicted PPI pairs increases. MCODE reached its peak around 9,000. The precision rate decreases as the network size increases. While the *F*-*value* increases at the beginning then goes down after reaching a peak. The increasing of *F*-*value* indicates that there are more true positive PPIs added to the network. The researchers can select different sizes of networks for various methods. The *F*-*value* of our method is higher than all the other methods when the size of network is larger than 10,000.

**Figure 13:**
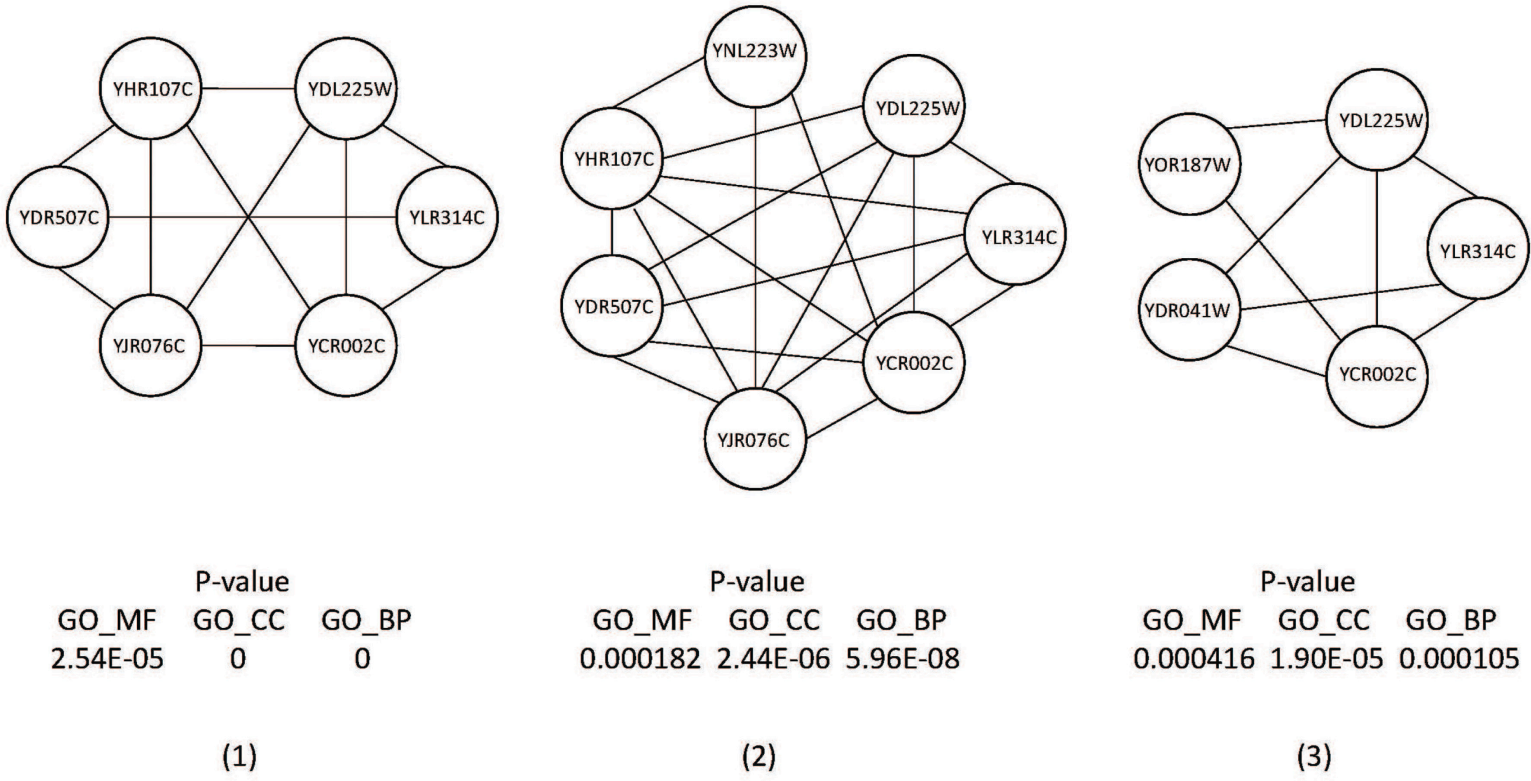
The false positive protein complexes which have low *P*-value and high local density.

Although some of our predicted complexes did not match any complexes in the benchmark complex set, we found that the predicted complexes have high biological significance and high local density as shown in Figure 13. They could be true complexes that are not discovered.

## 4. Conclusions

In this paper, we presented a Mutil-level PPINs reconstruction method (MLPR) for protein complex detection. Our method does not use the negative data, but only utilize the noisy existed database and incorporate more PPI evidences to reconstruct higher quality network. We mapped existing noisy data to multi-level networks and used the new level fingerprints similarity network to get high quality PPIs. Then we expanded the clusters from seed vertexes based on the reconstructed PPINs. The evaluation of our method indicates that our method achieved a higher *F*-*value* than other methods. In addition, our reconstructed PPI network significantly improves the performance of protein complex identification algorithms. Future work includes evaluation of individual features. We also plan to transfer our method to other link prediction research.

## Author contributions

BX conceived the study, participated in its design, carried out all experiments, and drafted the manuscript. YL drafted the manuscript. CL, JD, XL, and ZH reviewed the manuscript. CL conceived the study, participated in its design and coordination, and helped draft the manuscript. All authors read and approved the final manuscript.

### Conflict of interest statement

The authors declare that the research was conducted in the absence of any commercial or financial relationships that could be construed as a potential conflict of interest.
